# A Small and Slim Coaxial Probe for Single Rice Grain Moisture Sensing

**DOI:** 10.3390/s130303652

**Published:** 2013-03-14

**Authors:** Kok Yeow You, Hou Kit Mun, Li Ling You, Jamaliah Salleh, Zulkifly Abbas

**Affiliations:** 1 Communication Engineering Department, Faculty of Electrical Engineering, Universiti Teknologi Malaysia, Skudai, 81310 Johor, Malaysia; E-Mails: hkmun2@live.utm.my (H.K.M.); jsjamaliahsalleh@gmail.com (J.S.); 2 School of Allied Health Sciences, SEGi Kota Damansara, 47810 Selangor, Malaysia; E-Mail: llyou@segi.edu.my; 3 Department of Physics, Universiti Putra Malaysia, UPM Serdang, 43400 Selangor, Malaysia; E-Mail: za@science.upm.edu.my

**Keywords:** small open-ended coaxial probe, single rice grain, measured reflection coefficient, relative permittivity, gravimetric moisture content, transmission line, microwave measurement techniques

## Abstract

A moisture detection of single rice grains using a slim and small open-ended coaxial probe is presented. The coaxial probe is suitable for the nondestructive measurement of moisture values in the rice grains ranging from from 9.5% to 26%. Empirical polynomial models are developed to predict the gravimetric moisture content of rice based on measured reflection coefficients using a vector network analyzer. The relationship between the reflection coefficient and relative permittivity were also created using a regression method and expressed in a polynomial model, whose model coefficients were obtained by fitting the data from Finite Element-based simulation. Besides, the designed single rice grain sample holder and experimental set-up were shown. The measurement of single rice grains in this study is more precise compared to the measurement in conventional bulk rice grains, as the random air gap present in the bulk rice grains is excluded.

## 1. Introduction

Recently, there has been an increased interest in microwave measurement techniques for the determination of moisture content, *m.c.* (or dielectric properties) of bulk rice grains in the microwave frequencies range [1–5]. This is due to the fact that the moisture percentage (wet basis *m.c.*, ranging from 9% to 26%) in the rice grains plays an important role in the rice marketing and storage aspects. In marketing, the price of rice is dependent on the weight of the bulk rice, so accumulation of water in the rice grain will increase the price of rice. On the other hand, the moisture content in the grain mass determines the storage duration of the rice grains, in which *m.c.* < 13% indicates a storage duration of more than 60 days [6,7].

The *m.c.* of rice grains can be determined by either direct [8] or indirect methods [1–7]. Direct methods determine the *m.cd* by removing the moisture of the grains using oven drying methods (heating at 130 °C) or chemical reaction methods (extracting water using the reaction of iodine in sulfur dioxide). Both methods remove the moisture determine the true water content by the resulting weight loss. Indirect methods, in contrast, require the measurement of an electrical property of the grain using an instrument, a grain moisture meter. For very low frequency (DC) measurements, the desired electrical parameters are conductance, capacitance and resistance of the rice gain. The absorption power, resonant frequency, attenuation constant, reflection constant and transmission constant of the rice grain are the measured parameters of interest in microwave frequency measurements. Changes in electrical properties (or microwave properties) that can be directly correlated with a change in actual *m.c.* of the rice grain are obtained from the oven drying method (direct method). Recently, indirect methods have become more popular than direct methods due to their rapid tests and user friendly features.

The prime considerations in the measurement of grain moisture using indirect methods are the size of the instrument sensor, which is in direct contact with the rice grain samples and the accuracy of the measurements [5]. Various microwave waveguide methods were proposed for the above purpose, but some of those methods require specify dimensions of the rice grain bulk to fit inside the given size of the waveguide [2–4]. However, the rice grain bulk is composed of a mixture of air and rice grains. The random distribution of rice grains and the air gap causes a low repeatability and low precision in the measurements. Among the mentioned methods, an open-ended waveguide method is the simplest and a nondestructive way to measure the *m.c.* of rice grains. The measurement using commercial open-ended waveguides is suitable for a specific rice grains size (width and length). This is because the waves scattered from the waveguide aperture would penetratd through small single rice grains as the small rice grains faed to entirely cover the aperture area of commercial probes.

**Figure 1.**
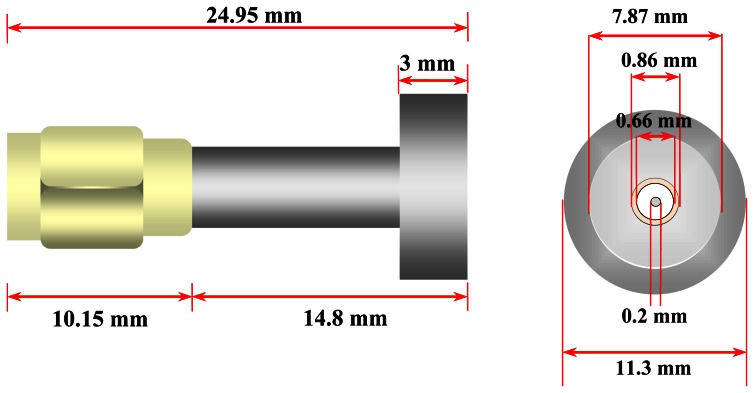
The side sectional view and front sectional view.

**Figure 2.**
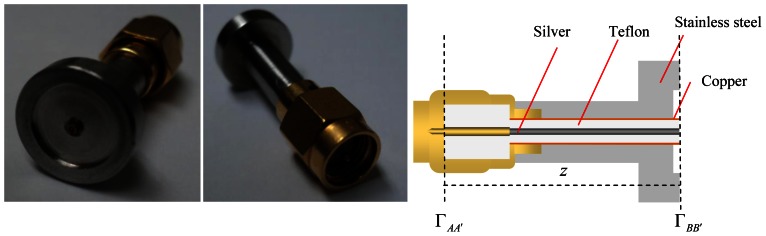
The Cross-sectional View.

In this study, a millimeter size slim open-ended coaxial probe has been fabricated to measure the moisture in small grains. The coaxial probe was fabricated from a 0.86 mm outer diameter (OD) semi-rigid coaxial cable equipped with a male-type SMA plug connector. The coaxial cable was machined flat and polished to form an open surface end. Then, the coaxial probe was protected and covered by a customized stainless steel sheath with a flange, as shown in Figures 1 and 2. Here, the main focus is put on the aperture of the probe placed against a rice grain sample ranging from 9% *m.c.* to 26% *m.c.* Typically, the open-ended coaxial probe is calibrated by using open air, short terminator and liquid load. Directivity error, source match error, and frequency tracking are corrected by this technique. Besides that, the rapid and simple calibration of the coaxial sensor without the use of short and load calibration kits were also proposed in this study (discussed in Section 3.2).

## 2. Configuration and Dimensions Coaxial Sensor

Figure 1 shows the side and the front sectional views of our milimeter size coaxial probe. The front sectional aperture of coaxial probe shows 2*a* = 0.20 mm diameter of inner conductor, 2*b* = 0.66 mm diameter of coaxial-filled Teflon and 0.86 mm diameter of the outer conductor. The coaxial-filled Teflon supports the coaxial line between the outer conductor and the inner conductor. Both inner and outer conductors guide the propagation wave in the coaxial line. In addition, an 11.3 mm total diameter steel flange is used to cover the total fringing field at the aperture probe. Figure 2 shows a picture and cross sectional structure of the coaxial probe.

## 3. Sensor Model

### 3.1. Reflection Coefficient Model

The reflection coefficient, Γ models of the millimeter coaxial probe are given as:

(1) $$\Gamma =\sum_{n=0}^{7}\delta_{n}{ɛ}_{r}^{n}f^{3}+\sum_{n=0}^{7}\beta_{n}{ɛ}_{r}^{n}f^{2}+\sum_{n=0}^{7}\gamma_{n}{ɛ}_{r}^{n}f+\sum_{n=0}^{7}\chi_{n}{ɛ}_{r}^{n}$$

where the symbols *δ* (in unit *f*^−3^), *β* (in unit *f*^−2^), *γ* (in unit *f*^−1^), and *χ* are the complex coefficients for the polynomial Equation (1). The symbol *f* represents the operation frequency.

The complex parameters in Equation (1) was obtained by fitting the polynomial coefficients with calculated values obtained from the Finite Element Method using the COMCOL simulator over a broad range of permittivity values. The complex polynomial coefficients with seven decimals for Equation (1) are listed in Table 1. Equation (1) is valid for small coaxial probes, satisfying the relative permittivity, *ε_r_* from 1 to 40 and the operation frequency from 0.4 GHz to 20 GHz. Comparison between the calculated and FEM simulated values for the reflection coefficient, Γ is shown in Table 2. If the FEM simulation results are used as the reference value, it is found that the percentage of relative error between both magnitudes of reflection coefficient will be less than 1%.

### 3.2. Calibration Model

In this study; the simplest technique of de-embedding of coaxial probe is by extending the transmission phase in which the phase of reflection coefficient at measurement plane; *AA*′ is extended towards the open end of coaxial probe; *BB*′ using exponential term of exp (*j2k_c_z*). First; a full one-port calibration technique was implemented at the *AA*′ plane using a commercial HP 85052D 3.5 mm calibration kit (open; short and load) which is only for network analyzer and cable error corrections. Secondly; under the assumption of quasi-TEM mode; the measured reflection coefficient; Γ_*AA*′_ of the sample at the plane *AA*′ can be de-embedded to the end of the probe connector which coincide with the calibration plane *BB*′ to give a reflection coefficient; Γ_*BB*′_ by [9]:

(2) $$\Gamma_{\text{BB}\prime }=\Gamma_{\text{AA}\prime }e^{2{\text{jk}}_{c}z}$$

where *z* and *k_c_*= (2*πf*/*c*)√*ε_c_* are the apparent physical length (in meter) and propagation constant of coaxial line, respectively. Symbols *f*, *c* and *ε_c_* are the operation measurement frequency (in Hz), velocity of light in free space (299792458 ms^−1^) and relative dielectric constant for the material filled in coaxial line (Teflon: *ε_c_* = 2.05), respectively. In this work, only “open” standard involved in the calibration, the reflection coefficient measurements, Γ_*Air*_ for air at the plane *AA*′ was taken, while the standard values for the air reflection coefficient, Γ_*Air_FEM*_ at the plane *BB*′ was simulated by Finite Element Method (COMSOL simulator). For the de-embedded process, the values of apparent physical length, *z* for coaxial line of the probe are required to determine. Once obtaining both values (Γ_*AA*′_ and Γ_*A FEM*_), the apparent physical length, *z* can be calculated as:

(3) $$\begin{array}{ll} z & =\left(\frac{-j}{2k_{c}}\right)\text{In}\left(\frac{\Gamma_{\text{Air}\_\text{FEM}}}{\Gamma_{\text{Air}}}\right)\\ & =z^{\prime }+jz^{\prime \prime } \end{array}$$

Simultaneously, the attenuation constant, *α* in the coaxial line can be found from the optimized length, *z* as:

(4) $$\alpha =\left(\frac{2\pi f}{c}\right)\left|\frac{z^{\prime \prime }}{z^{\prime }}\right|$$

**Figure 3.**
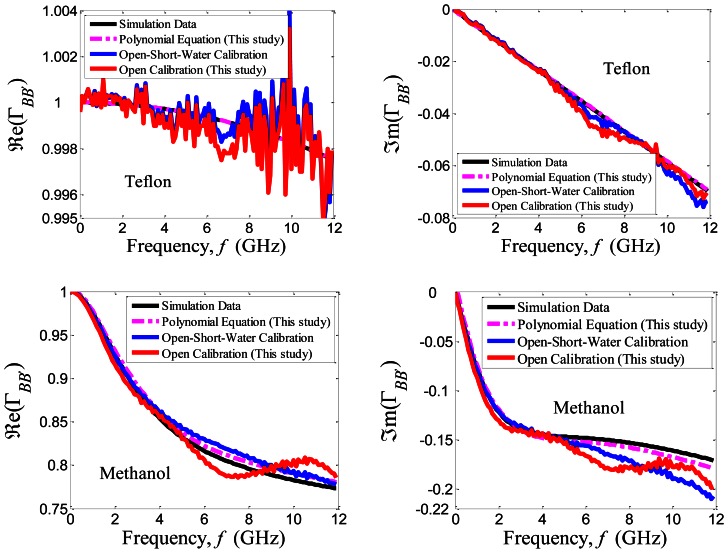
Variation in real and imaginary parts of reflection coefficient, ((image)e (Γ_*BB’*_) and ℑm (Γ_*BB’*_)) at plane *BB’* with frequency, *f* at (25 ± 1) °C.

Teflon and methanol liquid have been tested to verify the accuracy of the proposed calibration method. Figure 3 shows the comparison between the calibrated reflection coefficient measurement and the simulation results. In simulation, the relative permittivity, *ε_r_* of Teflon was 2.05. While, the relative permittivity, *ε_r_*, of methanol was computed by Cole-Cole model with parameters: *ε_s_* = 33.7, *ε_∞_* = 4.45, *τ* = 4.95 × 10^−11^ s and *α* = 0.036 [10]. The Cole-Cole model is:

(5) $${ɛ}_{r}={ɛ}_{\infty }+\frac{{ɛ}_{s}-{ɛ}_{\infty }}{1+{\left(j\omega \tau \right)}^{1-\alpha }}$$

In comparison, the calibrated measurement results using only the open standard have given a satisfy accuracy measurement up to 5 GHz. However, the noise inherent in both calibrated measurements are due to random errors contributed by the instruments or environmental measurement control. Moreover, the random errors are not taken into account in either calibration technique. The effect of the standing wave in the measurement of the open standard calibration becomes increasingly obvious when the operating frequency, *f*, is above 5 GHz.

The incident wave from the plane *AA*′ is transmitted to the plane *BB*′ by shifting phase of *k_c_z*, and is reflected back to input *AA*′ with the same shifting phase. Thus, the aperture reflection coefficient, Γ_*BB’*_ at the plane *BB’* can be found by the phase delay of 2*k_c_z* with respect to the measured Γ_*AA’*_ at the plane *AA*′ and the transmission line relationship is given as in Equation (2). However, the transmission line is imperfect and a fringing field occurs near the aperture probe. Hence, a phase shift between the forward wave and the reflected wave occurs, and produces the standing wave due to the superposition between the incident wave and the reflected wave inside the coaxial line [11]. The standing wave effect can be ignored if the operation quarter wave length, *λ*/4 in the coaxial line is large than the physical length, *z* of coaxial line. For instance, 5 GHz of operation frequency will give *λ*/4 = 15 mm which is smaller than the physical length, *z* ≈ 22 mm, thus, the standing wave effect was significant when the operation frequency, *f*, was increased.

### 3.3. Inverse Model

For inverse solutions, the predicted values of the relative dielectric constant, *ε_r_*,’ of a rice grain sample is obtained by minimizing the difference between the measured reflection coefficient, Γ_*BB’*_ and Equation (1), Γ by referring to the trial function, *ψ*:

(6) $$\Psi =\sum_{1}^{\text{Data}}(\frac{ℑm(\Gamma )}{ℜe(\Gamma )}-m\frac{ℑm(\Gamma_{\text{BB}\prime })}{ℜe(\Gamma_{\text{BB}\prime })})$$

**Figure 4.**
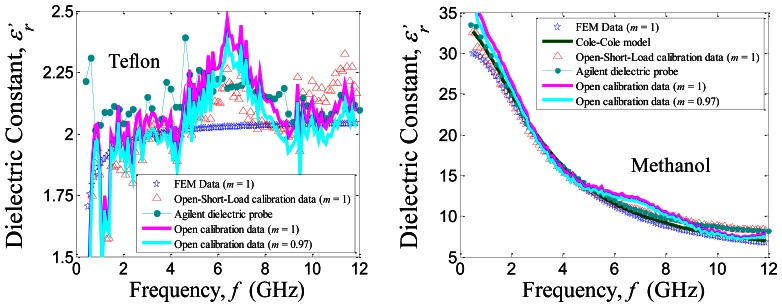
Variation in dielectric constant, ${ɛ}_{r}^{\prime }$ with frequency, *f* for Teflon and liquid methanol at (25 ± 1) °C.

Finding the zero routine was realized using the MATLAB fzero command. The initial approximate value in the numerical prediction was equal to 5 – *j* 0.001. The *m* terms in Equation (6) denote the weighted parameter for ratio of $\frac{ℑm(\Gamma_{\text{BB}\prime })}{ℜe(\Gamma_{\text{BB}\prime })}$. The weighted parameter, *m* is suggested due to the measurement with calibration using Equation (2) (only open standard: *ε_r_* = 1) does not consider the fringing effects for the medium loss samples. The predicted dielectric constant, ${ɛ}_{r}^{\prime }$ for Teflon and methanol liquid over frequencies 0.5 GHz to 12 GHz at room temperature (25 °C) are validated and compared with the FEM simulation, Cole-Cole models and the Agilent 85070E dielectric probe as shown in Figure 4. The diameter of outer radius for the Agilent 85070E probe (2*b* = 3 mm) is approximately 4.5 times larger than the studied coaxial probe.

## 4. Experimental

The measurement reflection coefficient using millimeter coaxial probe that consists of the Agilent E5071C network analyzer in the frequency range between 0.5 GHz to 12 GHz was carried out at room temperature. The open end of the coaxial line was terminated by single rice grain sample. Normally, the length and width for various kinds of rice grains was in the range of 4.8–7.8 mm and 1.5–2.8 mm, respectively. Thus, the fringing field (sensing area ≈ 2*b*) [9] from the coaxial probe aperture was sufficiently covered by the single rice sample. This can be applied based on the principle that the different signals are reflected from the terminal surface of the moist grain through the coaxial opening.

*Jati*™ long grain white rice grown on the fertile soil of Kedah, the Rice Bowl of Malaysia, was used as the experimental sample. The rice grains were divided into different groups of 200 g per group. Each group of grains was sprayed with different estimated quantities of distilled water to achieve desired moisture levels. The bulk grains in each respective group were stirred and sealed in a container at 4 °C for 72 h to ensure a uniform water distribution within the bulk grains. The grains were conditioned to room temperature for 10 h prior to the measurements. Finally, 10 g of each group of bulk grain rice was dried in an air convection oven at 130 °C for 24 h [8]. The average moisture content, *m.c.* (in unit %) of each group of bulk rice grain was calculated on a wet basis as:

(7) $$m.c(\%)=\frac{m_{\text{water}}}{m_{\text{water}}+m_{\text{dry bulk grain}}}\times 100$$

where *m_water_* and *m_dry bulk grain_* are mass of water and dry bulk grain, respectively.

**Figure 5.**
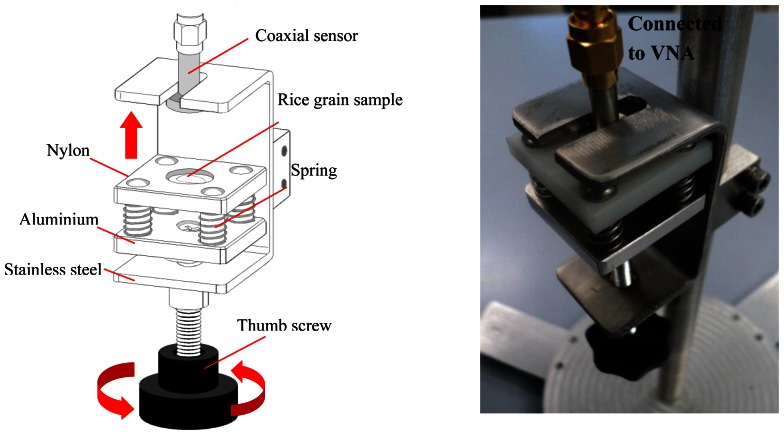
Experimental Set-up.

In this measurement, a specific holder was customized for the measurement of a selected single rice grain. The customized holder has a movable nylon platform which was mounted on a retort stand as shown in Figure 5. A single rice grain was randomly selected from each 10 g of bulk grain and placed into a narrow and depth concave surface at the center of the platform. The steel flange of the coaxial probe was rigidly entered from the holder edge into a narrow space interval. The rice grain on the nylon platform was moved forward to the aperture coaxial probe by using a thumb screw. The concave circular surface on platform was used as a probe guide to ensure that the probe aperture is exactly touching the rice sample. The four springs were employed to ensure that the aperture probe contacts firmly with the interface single rice grain and to avoid the errors in the measurement of interface air gaps.

## 5. Results and Discussion

**Figure 6.**
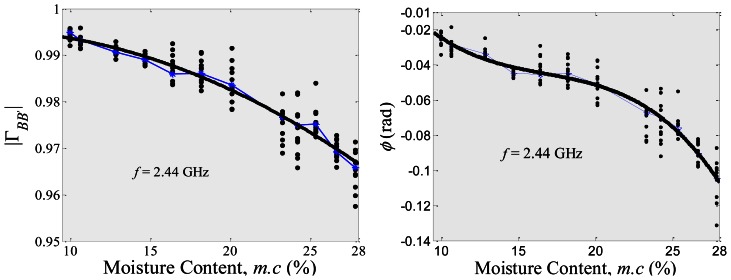

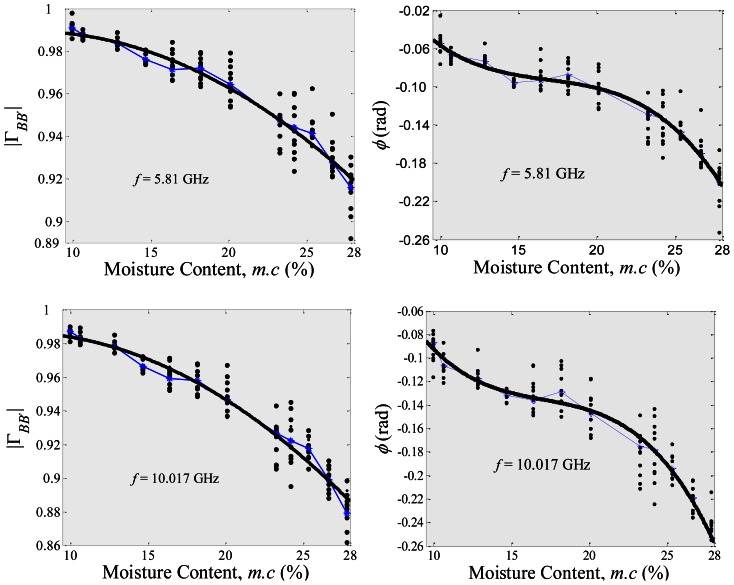
The relationship between reflection coefficient (magnitude, Γ_*BB’*_ and phase, *ø*) and the moisture content, *m.c* at 2.44 GHz, 5.81 GHz and 10.02 GHz, respectively.

Figure 6 shows the reflection coefficient, Γ_*BB′*_, for 10 selected single rice grains from a bulk rice grain sample, where the bulk has a certain average water content, measured using the millimeter coaxial probe. As known, the *m.c.* for a bulk rice grain sample (which consists of thousands of single rice grains) is a statistical mean value due to the slightly different *m.c_single_* of each single grain. Thus, the deviation of the actual *m.c_single_* distribution in a single rice grain is higher as compared to the average *m.c.* of the entire bulk rice sample. This will lead to a scattered measurement of the reflection coefficient, Γ_*BB’*_, of the single grain in referring to the average *m.c.* of the bulk sample, as shown in Figure 6. However, Figure 6 does show a significant change of the measured reflection coefficient, Γ_*BB’*_ of a single rice grain with the *m.c.* of bulk rice grain. The black solid line in Figure 6 is a regression fitting line from the average of measured reflection coefficient data (blue point-line). The average of the measured reflection coefficients was obtained from the 10 data points of the measured reflection coefficients for each bulk moisture content, *m.c.* The rice moisture calculations are based on a gravimetric method, thus differential effects of density between the bulk rice and the single rice grain in the measurement were ignored. In this study, the 2.44 GHz and the 5.81 GHz frequencies were chosen due to the fact those frequencies correspond to a free unlicensed band which is specifically for industrial, scientific and medical (ISM) measurement purposes. The 10.02 GHz band was also chosen, since the dielectric loss for the water is greater at around 10 GHz and thus provides a comparative approach for the rice measurements at higher frequencies.

The polynomial regressions for the reflection coefficient, Γ_*BB’*_ (magnitude, |Γ_*BB’*_| and phase, *ø*) with respect to *m.c.* (in % units), are given in Equation (8) as listed in Table 3. Subsequently, Equation (8) was used by the coaxial probe to predict the *m.c.* (in % units), in a rice grain. It was found that, at higher frequencies, the point of measured reflection coefficient, Γ_*BB’*_ shows a large variation of *m.c.* (in % units) of rice grains.

**Figure 7.**
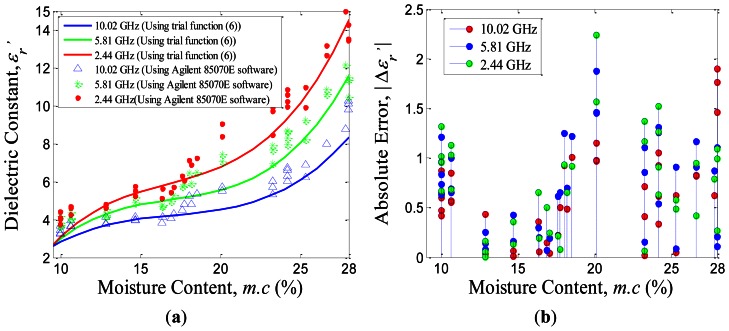
(**a**) The variations in ${ɛ}_{r}^{\prime }$ of single rice grain with its bulk moisture content, *m.c.* at 2.44 GHz, 5.81 GHz and 10.02 GHz, respectively. (**b**) The absolute deviation, $\left|\Delta {ɛ}_{r}^{\prime }\right|$ of dielectric constant prediction between the use of studied inversion technique and the Agilent 85070E software computation.

The variations in relative dielectric constant, ${ɛ}_{r}^{\prime }$, of rice grains with the percentage of *m.c.* at 2.44 GHz, 5.81 GHz and 10.02 GHz, respectively, are plotted in Figure 7(a). The solid line of relative dielectric constant, ${ɛ}_{r}^{\prime }$, in Figure 7(a) was the inverse of the reflection coefficient and refers to the trial function of Equation (6) with *m* = 1. The real part, ℜe(Γ_*BB’*_) and imaginary part, 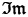
(Γ_*BB’*_) in Equation (6) were calculated by using relationship of ℜe(Γ_*BB’*_)+*j* 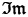
(Γ_*BB’*_)=|Γ_*BB’*_|exp(*jØ*), where the values of |Γ_*BB’*_| and *Ø* were obtained from Equation (8), while, the reference values of ℜe(Γ) and 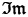 (Γ) in Equation (6) were computed using Equation (1). The measurement of dielectric constant points, ${ɛ}_{r}^{\prime }$, of the single rice grain in Figure 7(a) were carried out using the studied coaxial probe with the Agilent 85070 E.06.01.36 software in order to verify the data obtained from the dielectric inversion work. The tolerance, $\left|\Delta {ɛ}_{r}^{'}\right|$ of dielectric constant prediction between both techniques is shown in Figure 7(b), which gives the maximum deviation, $\left|\Delta {ɛ}_{r}^{\prime }\right|$ ≈ 2 for all frequencies ranging 9.5% *m.c.* to 28% *m.c.*

## 6. Conclusions

The proposed coaxial probe has a small sensing area which covers the size of single rice grain and provides a nondestructive and real time moisture measurement for single rice grains. Moreover, the single grain measurement does not depend on the bulk density of the rice grains, thus the uncertainty of bulk density in the rice measurement (due to different rates of broken rice in the bulk grain) can be ignored. In this study, moisture and dielectric models were created to suit the studied coaxial probe. The proposed simple de-embedding technique provides a rapid and low cost calibration procedure for the coaxial probe. However, the technique does not consider the systematic and random noises along the coaxial line, and thus it is suitable for a short coaxial probe, (*z*<*λ*/4) due to the significant standing wave that is produced inside the long coaxial line.

## Figures and Tables

**Table 1.** Complex Coefficient for Equation (1).

Complex Coefficients in Equation (1)							
*δ_0_*	−1.2489993 × 10^−42^ −*j*2.8394016 × 10^−42^	*β_0_*	4.068759 × 10^−32^ + *j*6.0446093 × 10^−32^	*γ_0_*	−2.3422556 × 10^−22^ − *j*1.0834847 × 10^−21^	*χ_0_*	3.2710002 × 10^−13^ + *j*4.3278983 × 10^−13^
*δ _1_*	2.8571222 × 10^−40^ + *j*3.768492 × 10^−40^	*β_1_*	−8.0370086 × 10^−30^ − *j*7.583469 × 10^−30^	*γ_1_*	4.4984192 × 10^−20^ + *j*1.5879853 × 10^−19^	*χ_1_*	−6.0520681 × 10^−11^ − *j*5.0636272 × 10^−11^
*δ_2_*	−2.3273679 × 10^−38^ − *j*1.5437565 × 10^−38^	*β_2_*	5.7873753 × 10^−28^ + *j*2.8089148 × 10^−28^	*γ_2_*	−3.0910003 × 10^−18^ − *j*9.0522701 × 10^−18^	*χ_2_*	4.0103924 × 10^−9^ + *j*1.6441936 × 10^−9^
*δ_3_*	7.6388153 × 10^−37^ + *j*6.415406 × 10^−38^	*β_3_*	−1.6900591 × 10^−26^ − *j*8.6931643 × 10^−28^	*γ_3_*	8.3505821 × 10^−17^ + *j*2.6457801 × 10^−16^	*χ_3_*	−1.0502173 × 10^−7^ + *j*7.2034269 × 10^−10^
*δ_4_*	-6.5374999 × 10^−36^ + *j*5.4983527 × 10^−36^	*β_4_*	1.7398403 × 10^−25^ − *j*3.1661421 × 10^−26^	*γ_4_*	−7.5782519 × 10^−16^ − *j*5.0073506 × 10^−15^	*χ_4_*	9.5824766 × 10^−7^ − *j*2.7560669 × 10^−7^
*δ_5_*	2.124032 × 10^−35^ − *j*1.2607055 × 10^−35^	*β_5_*	−4.0862657 × 10^−24^ + *j*1.4318571 × 10^−25^	*γ_5_*	3.2419268 × 10^−15^ + *j*6.0109573 × 10^−14^	*χ_5_*	−4.2081991 × 10^−6^ + *j*1.338741 × 10^−6^
*δ_6_*	−6.9741684 × 10^−35^ − *j*9.6327922 × 10^−36^	*β_6_*	−2.122961 × 10^−25^ − *j*4.29076 × 10^−25^	*γ_6_*	−8.983224 × 10^−15^ − *j*2.9066269 × 10^−12^	*χ_6_*	1.1443542 × 10^−5^ − *j*3.7209467 × 10^−6^
*δ_7_*	5.6295484 × 10^−35^ − *j*1.6824543 × 10^−35^	*β_7_*	−1.1659675 × 10^−25^ + *j*2.7664167 × 10^−25^	*γ_7_*	7.1748551 × 10^−15^ − *j*7.8926933 × 10^−14^	*χ_7_*	9.9999087 × 10^−1^ + *j*2.5383631 × 10^−6^

**Table 2.** Calculated and Simulated Reflection Coefficient, Γ.

*f* (GHz)	Reflection Coefficient, Γ(*ε_r_=* 1 – *j* 0)		Relative Error (%)	Reflection Coefficient, Γ(*ε_r_=*40 – *j* 5)		Relative Error (%)
	Equation (1)	Simulations		Equation (1)	Simulations	
1	0.9999955− *j* 0.002931223	0.9999957− *j* 0.002926868	0.00002	0.9855305− *j* 0.09609176	0.9824686− *j* 0.10045476	0.2646
10	0.9995828− *j* 0.02934018	0.9995701− *j* 0.02929747	0.00139	0.5257350− *j* 0.7289402	0.5155215− *j* 0.7370167	0.0745
18	0.9986448− *j* 0.05295265	0.9985924− *j* 0.05287694	0.00563	0.02905127− *j* 0.8579390	0.02131934− *j* 0.85924107	0.12505

**Table 3.** Polynomial functions for the reflection coefficient magnitude, |Γ_*BB’*_|, and phase, *ø*, (in rad units) respect to moisture content, *m.c.*, of the bulk rice (in unit%)

For *f* = 2.44 GHz, |Γ_*BB*_’| = −4.918756 × 10^−5^* m.c*^2^ + 3.694446 × 10^−4^*m.c* + 0.9948964, (8a) $R^{2}=0.98420$ *Ø* = −3.043213 × 10^−5^ *m.c*^3^ + 1.530463 × 10^−3^ *m.c*^2^ − 2.729901 × 10^−2^*m.c* + 0.1257916, (8b) $R^{2}=0.99338$
For *f* = 5.81 GHz, |Γ_*BB*′_| = −1.616480 × 10^−4^ *m.c*^2^ + 2.323330 × 10^−3^ *m.c* + 0.9810024, (8c) $R^{2}=0.98386$ *Ø* = −6.330536 × 10^−5^*m.c*^3^ + 3.209160 × 10^−3^*m.c*^2^ − 5.643064 × 10^−2^*m.c* + 0.2498354, (8d) $R^{2}=0.98802$
For *f* = 10.07 GHz, |Γ_*BB*′_| = −2.135387 × 10^−4^ *m.c*^2^ + 2.691858 × 10^−3^* m.c* + 0.9783604, (8e) $R^{2}=0.98177$ *Ø* = −7.264877 × 10^−5^*m.c*^3^ + 3.722521 × 10^−3^*m.c*^2^ − 6.603589 × 10^−2^*m.c* + 0.2683548, (8f) $R^{2}=0.99068$

• The symbol *m.c* refers to the percentage of bulk rice moisture content.
